# Polyfunctional T follicular helper cells drive checkpoint-inhibitor diabetes and are targeted by JAK inhibitor therapy

**DOI:** 10.1172/jci.insight.188843

**Published:** 2025-07-08

**Authors:** Nicole L. Huang, Jessica G. Ortega, Kyleigh Kimbrell, Joah Lee, Lauren N. Scott, Esther M. Peluso, Sarah J. Wang, Ellie Y. Kao, Kristy Kim, Jarod Olay, Jaden N. Nguyen, Zoe Quandt, Trevor E. Angell, Maureen A. Su, Melissa G. Lechner

**Affiliations:** 1 Division of Endocrinology, Diabetes, and Metabolism, UCLA David Geffen School of Medicine, Los Angeles, California, USA.; 2 UCSF Medical School, San Francisco, California, USA.; 3 University of Kansas Medical School, Kansas City, Kansas, USA.; 4 UCLA/California Institute of Technology Medical Scientist Training Program, UCLA David Geffen School of Medicine, Los Angeles, California, USA.; 5 California State Polytechnic University, Pomona, California, USA.; 6 Department of Microbiology, Immunology, and Molecular Genetics, UCLA David Geffen School of Medicine, Los Angeles, California, USA.; 7 Division of Endocrinology and Metabolism, UCSF Medical School, San Francisco, California, USA.; 8 Division of Endocrinology and Diabetes, University of Southern California Keck School of Medicine, Los Angeles, California, USA.; 9 Division of Pediatric Endocrinology, UCLA David Geffen School of Medicine; Los Angeles, California, USA.

**Keywords:** Autoimmunity, Oncology, Autoimmune diseases, Cancer immunotherapy, Diabetes

## Abstract

Immune checkpoint inhibitors (ICI) have revolutionized cancer therapy, but their use is limited by the development of autoimmunity in healthy tissues as a side effect of treatment. Such immune-related adverse events (IrAE) contribute to hospitalizations, cancer treatment interruption, and even premature death. ICI-induced autoimmune diabetes mellitus (ICI-T1DM) is a life-threatening IrAE that presents with rapid pancreatic β-islet cell destruction leading to hyperglycemia and life-long insulin dependence. While prior reports have focused on CD8^+^ T cells, the role for CD4^+^ T cells in ICI-T1DM is less understood. We identify expansion of CD4^+^ T follicular helper (Tfh) cells expressing IL-21 and IFN-γ as a hallmark of ICI-T1DM. Furthermore, we show that both IL-21 and IFN-γ are critical cytokines for autoimmune attack in ICI-T1DM. Because IL-21 and IFN-γ both signal through JAK/STAT pathways, we reasoned that JAK inhibitors (JAKi) may protect against ICI-T1DM. Indeed, JAKi provide robust in vivo protection against ICI-T1DM in a mouse model that is associated with decreased islet-infiltrating Tfh cells. Moreover, JAKi therapy impaired Tfh cell differentiation in patients with ICI-T1DM. These studies highlight CD4^+^ Tfh cells as underrecognized but critical mediators of ICI-T1DM that may be targeted with JAKi to prevent this grave IrAE.

## Abstract 2

Multifunctional IL-21^+^ IFNg^+^ CD4^+^ T follicular helper cells are key drivers of checkpoint immunotherapy-induced autoimmune diabetes that can be targeted with JAK inhibitor therapy.

## Graphical abstract

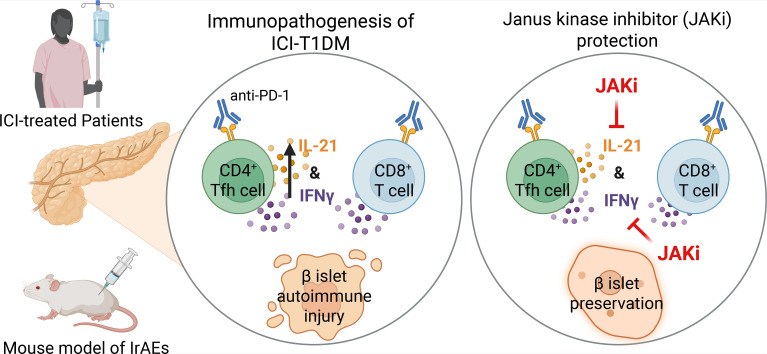

## Introduction

Immune checkpoint inhibitor (ICI) therapies have significantly improved outcomes for patients with many types of advanced cancers. However, their use is limited by the development of autoimmune toxicities in healthy tissues in nearly two-thirds of patients (1–3). ICI-induced autoimmune diabetes mellitus (ICI-T1DM) is a rare but life-threatening immune-related adverse event (IrAE) that occurs in 1%–2% of patients treated with ICI (4). ICI-T1DM presents as a rapidly progressive autoimmune destruction of pancreas β-islet cells, accompanied by hyperglycemia and often ketoacidosis (4–6). Patients with ICI-T1DM have permanent pancreatic endocrine insufficiency and require life-long insulin replacement therapy. In patients receiving ICI therapy for advanced malignancies, this additional comorbidity can add another debilitating and overwhelming layer of complexity to their care. On the other hand, in the growing number of patients who receive ICI therapy for early stage or curable disease, ICI-T1DM represents a permanent sequela of treatment that can negatively affect quality of life long after cancer resolution.

Currently, no therapies exist to prevent endocrine IrAEs, including ICI-T1DM (4, 5, 7–9). Understanding immune mechanisms that drive autoimmunity may identify therapeutic targets to reduce IrAEs. We recently identified IL-21^+^ T follicular helper (Tfh) cells as critical mediators of ICI-thyroiditis (10), another common endocrine IrAE seen in 15%–25% of patients treated for ICI. Like ICI-T1DM, ICI-thyroiditis presents as brisk autoimmune destruction of thyroid gland cells and loss of thyroid function over a period of weeks (9, 11). We found that thyrotoxic IFN-γ^+^CD8^+^ T cells in the thyroid were driven by IL-21 from CD4^+^ Tfh cells and inhibition of IL-21 prevented ICI-thyroiditis (10). Whether Tfh cells contribute to the development of ICI-T1DM and may be therapeutically targeted to reduce pancreas autoimmunity during ICI therapy has not yet been explored.

In addition to developing mechanism-based therapies for IrAEs, a practical consideration is the urgent need for near-term strategies to reduce autoimmunity in the many patients currently receiving ICI therapy. As clinical indications for ICI therapy expand (12), the number of patients with IrAEs will surge — as will the need for therapies to halt severe or life-threatening autoimmune toxicities like ICI-T1DM. Janus kinase inhibitors (JAKi) are a class of orally bioavailable medications now widely used to treat spontaneous autoimmune diseases like alopecia, psoriasis, and arthritis (13–15). These agents block JAK signaling, which is required for many T cell cytokine responses (13). Indeed, Waibel et al. (16) reported preservation of β-islet cell function and decreased insulin requirements in individuals with spontaneous T1DM in a phase 2 trial of JAKi baricitinib. However, the potential of JAKi to halt the rapid and often fulminant autoimmune responses seen in IrAEs has only been explored recently. JAK1/2 inhibitor ruxolitinib notably improved survival from 3.4% to 60% in a cohort of patients with steroid refractory ICI-myocarditis, another rare but deadly IrAE, when given in combination with CTLA-4 agonist abatacept (17). Based upon their promise in spontaneous autoimmune diseases and ICI-myocarditis, we hypothesized that JAKi could be utilized to prevent endocrine IrAEs.

In this study, we identify multifunctional CD4^+^ Tfh cells expressing IL-21 and IFN-γ as antigen-specific mediators of autoimmune tissue injury in ICI-T1DM. Furthermore, we show that both IL-21 and IFN-γ are critical cytokines in autoimmune attack during ICI-T1DM and that inhibition of these cytokine pathways by JAKi therapy can prevent ICI-T1DM. Moreover, we show that JAKi treatment decreases islet-infiltrating Tfh cells in a mouse model of IrAEs and Tfh cell differentiation in patients with ICI-T1DM. These studies highlight CD4^+^ Tfh cells as underrecognized but critical mediators of ICI-T1DM that may be targeted with JAKi to prevent this life-threatening endocrine IrAE.

## Results

### Individuals with ICI-T1DM have increased Tfh cell responses.

Tfh cells contribute to multiple spontaneous autoimmune diseases, including T1DM (18, 19), where they can signal to B cells in germinal centers and promote pathogenicity of CD8^+^ T cells (10, 18–21). Expansion of Tfh cells has recently been linked to the development of IrAEs in patients treated for ICI. Herati et al. (22) reported an increase in circulating Tfh cells after influenza vaccination in anti–PD-1–treated patients who went on to develop IrAEs. Furthermore, in individuals with ICI-thyroiditis, IL-21^+^ CD4^+^ Tfh cells are key drivers of thyroid autoimmune attack (10). Therefore, we hypothesized that Tfh cells may also contribute to the development of ICI-T1DM.

**Figure 1:**
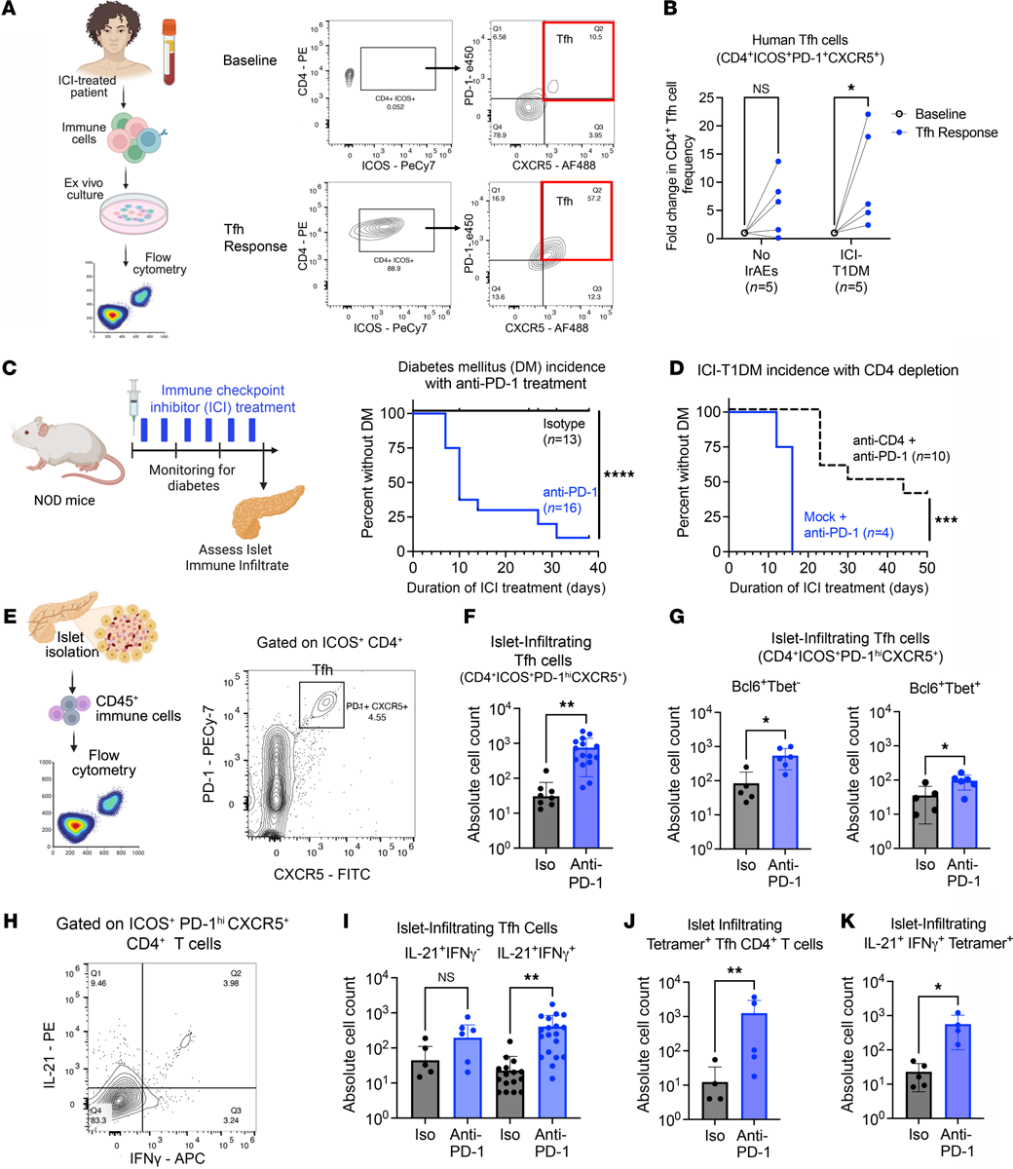
Increased CD4^+^ Tfh cell response in individuals with ICI-T1DM and a mouse model of IrAEs. (**A**) Representative flow cytometry of PBMC from ICI-treated patients at baseline and after ex vivo culture under Tfh-skewing conditions (23). (**B**) Fold change in Tfh cell frequency for individuals with ICI-T1DM versus ICI-treated individuals with no irAEs. Each pair represents 1 individual. (**C**) DM incidence in NOD mice treated with anti–PD-1 (8 males [M]/8 females [F]) or isotype (Iso) (6M/7F). (**D**) DM incidence in anti–PD-1 treated NOD mice with a depleting anti-CD4 antibody (5M/5F) or isotype (Mock) (2M/2F). (**E**) Representative flow cytometry for islet-infiltrating Tfh cells. (**F**) Quantification of Tfh cells (CD4^+^ICOS^+^PD-1^hi^CXCR5^+^) within islets of anti–PD-1–treated (*n* = 16) versus Iso-treated (*n* = 8) mice. (**G**) Quantification of Bcl6^+^Tbet^–^ and Bcl6^+^Tbet^+^ subsets within CD4^+^ICOS^+^PD-1^hi^CXCR5^+^ cells in the islets of anti–PD-1–treated (*n* = 6) versus Iso-treated (*n* = 5) mice. (**H**) Representative flow cytometry and quantification of islet-infiltrating IL-21– and IFN-γ–producing Tfh cells in Iso-treated (*n* = 7) and anti–PD-1–treated (*n* = 13) mice. (**I**) Quantification of IL-21^+^IFN-γ^–^ and IL-21^+^IFN-γ^+^ subsets within CD4^+^ ICOS^+^PD-1^hi^CXCR5^+^ cells in the islets of anti–PD-1–treated (*n* = 19) versus Iso-treated (*n* = 8–9) mice. (**J**) Quantification of BDC2.5-mimotope tetramer^+^ Tfh cells within the islets of Iso-treated (*n* = 6) versus anti–PD-1–treated (*n* = 5) mice. (**K**) Comparison of islet-infiltrating IL-21^+^IFN-γ^+^tetramer^+^CD4^+^ Tfh cells between anti–PD-1–treated (*n* = 4) and Iso-treated (*n* = 5) mice. Each point represents data from 1 animal, and data are presented as mean ± SD. Comparisons by 2-way ANOVA for paired samples with subsequent pairwise comparisons (**B**), log-rank test (**C** and **D**), Welch’s *t* test (**G**), Brown-Forsythe and Welch ANOVA (**I**), or Mann-Whitney *U* test (**F**, **J**, and **K**); *P < 0.05, **P < 0.01, ***P < 0.001, ****P < 0.0001.

To test this idea, we evaluated Tfh cells (CD4^+^ICOS^+^PD-1^hi^CXCR5^+^) in peripheral blood specimens from patients with ICI-T1DM versus patients who received ICI therapy but did not develop IrAEs. Because prior work showed that Tfh cell response, but not baseline levels of circulating Tfh cells, was predictive of IrAEs, we compared the magnitude of Tfh cell expansion between groups after Tfh skewing ex vivo (23) (Figure 1A). Indeed, patients with ICI-T1DM had a more robust Tfh cell response than those without IrAEs, with increased CD4^+^ICOS^+^PD-1^hi^CXCR5^+^ cells compared with controls without autoimmunity (Figure 1B; *P* < 0.05). These data suggest that individuals with ICI-T1DM have increased CD4^+^ Tfh cell responses compared with individuals who do not develop IrAEs.

### Antigen-specific IL-21^+^IFN-γ^+^CD4^+^ Tfh cells are increased in the pancreatic islets of mice with ICI-T1DM.

To better understand the role of Tfh cells in the immunopathogenesis of ICI-T1DM in vivo, we then used a mouse model of IrAEs. Previously, we reported the development of multiorgan immune infiltrates in autoimmunity-prone nonobese diabetic (NOD) mice following ICI treatment, including thyroiditis, colitis, and accelerated DM (10, 24). As expected, male and female NOD mice (7–9 weeks of age) treated with continued cycles of anti–programmed death 1 (anti–PD-1) antibody (10 mg/kg/dose, twice weekly), developed ICI-T1DM at a median of 10 days, while isotype-treated controls remained healthy after 4 weeks (Figure 1C; *P* < 0.0001).

T cells play a key role in the development of IrAEs in multiple tissues (10, 25–28), including the pancreas (4, 29–31). As expected, NOD mice with genetic deletion of TCRα, which leads to an absence of mature CD4^+^ and CD8^+^ T cells, were completely protected from ICI-T1DM (Supplemental Figure 1A; supplemental material available online with this article; https://doi.org/10.1172/jci.insight.188843DS1). Prior studies have demonstrated the importance of IFN-γ–producing CD8^+^ T cells in mouse models of ICI-T1DM (29–31). On the other hand, the role of CD4^+^ T cells has been less explored but is important in other IrAEs (10, 24, 32–34). Additionally, because CD4^+^ T cell responses may not be as central to ICI antitumor efficacy, they might be therapeutic targets to reduce IrAEs in patients with cancer while preserving efficacy (24, 32, 35).

Antibody depletion of CD4^+^ T cells in ICI-treated WT NOD mice significantly delayed the onset of autoimmune diabetes (Figure 1D and Supplemental Figure 1B), suggesting a CD4^+^ T cell contribution to ICI-T1DM disease progression. We then compared the frequency of CD4^+^ Tfh cells within pancreatic islets of NOD mice after 3 weeks of anti–PD-1 or isotype control therapy (Figure 1E). Indeed, anti–PD-1–treated mice had increased islet-infiltrating Tfh cells (CD4^+^ICOS^+^PD-1^hi^CXCR5^+^) compared with isotype controls (Figure 1F,]; *P* < 0.01) — a trend toward increased Tfh cells was also found in pLN — but this difference was not statistically significant (Supplemental Figure 1C). Within this putative ICOS^+^PD-1^hi^CXCR5^+^CD4^+^ Tfh cell population, we identified both Bcl6^+^Tbet^–^ Tfh and Bcl6^+^Tbet^+^ Tfh-like subsets that were increased within the pancreatic islets of anti–PD-1–treated mice (Figure 1G; *P* < 0.05 for both). We next evaluated IL-21 and IFN-γ cytokine production by islet-infiltrating Tfh cells by flow cytometry (Figure 1H). Dual producing IL-21^+^IFN-γ^+^ Tfh cells were increased within the islets of anti–PD-1 treated mice (Figure 1I, *P* < 0.01), with a nonsignificant trend within the islets for increased IL-21^+^IFN-γ^–^ Tfh cells (Figure 1I, *P* = ns). Such multifunctional IL-21^+^IFN-γ^+^ Tfh CD4^+^ cells have previously been described as mediators of immune response in spontaneous autoimmune diseases (e.g., lupus and peripheral neuropathy) and viral infections (36–39). In summary, anti–PD-1 treatment and development of ICI-T1DM is associated with islet infiltration by Bcl6^+^Tbet^–^ Tfh and Bcl6^+^Tbet^+^ Tfh-like cells expressing IL-21 and IFN-γ.

In spontaneous T1DM, autoimmune Tfh cells classically reside within pancreatic lymph nodes (pLN) and can traffic into inflamed islets (40, 41). While we did not observe a significant increase in ICOS^+^ PD-1^hi^CXCR5^+^CD4^+^ Tfh cells within pLN following anti–PD-1 therapy (Supplemental Figure 1C), ICI treatment increased Tfh cell expression of chemokine receptors associated with trafficking to inflamed pancreatic islets, including CXCR6 (29, 30, 41) (Supplemental Figure 1D). Indeed, islet-infiltrating IL-21^+^IFN-γ^+^ Tfh cells expressed CXCR6^+^ that was increased with anti–PD-1 therapy (Supplemental Figure 1E). These data suggest that pLN Tfh cells may upregulate CXCR6 and migrate to the islet in response to ICI treatment.

We then wondered whether islet-infiltrating IL-21^+^IFN-γ^+^ Tfh cells were autoantigen specific or responding as bystanders to the inflamed islet. Fife and colleagues previously established a pathogenic role for BDC2.5-mimotope CD4^+^ T cells in NOD mice with accelerated autoimmune DM due to loss of PD-1 (42). Therefore, we used an MHC class II BDC2.5 tetramer to quantify autoantigen-specific CD4^+^ T cells in our mouse model. Twenty-seven percent of islet-infiltrating BDC2.5-mimotope tetramer^+^CD4^+^ T cells (hereafter referred to as tetramer^+^) had a surface phenotype consistent with Tfh cells (ICOS^+^PD-1^hi^CXCR5^+^) by flow cytometry (Supplemental Figure 2, A and B), expressed canonical Tfh transcription factor Bcl6 (Supplemental Figure 2, C and D), and produced cytokines IL-21 and IFN-γ (Supplemental Figure 2, E and F). Furthermore, anti–PD-1–treated mice had more tetramer^+^ CD4^+^ Tfh cells in pancreatic islets compared with isotype-treated controls (Figure 1J; *P* < 0.01), and these cells showed high dual expression of IL-21 and IFN-γ (Figure 1K; *P* < 0.05). Taken together, these data support a role for antigen-specific, polyfunctional IL-21^+^IFN-γ^+^CD4^+^ Tfh cells in the autoimmune attack on pancreas β-islet cells during ICI therapy.

### IL-21 and IFN-γ are important cytokine mediators of ICI-T1DM.

**Figure 2:**
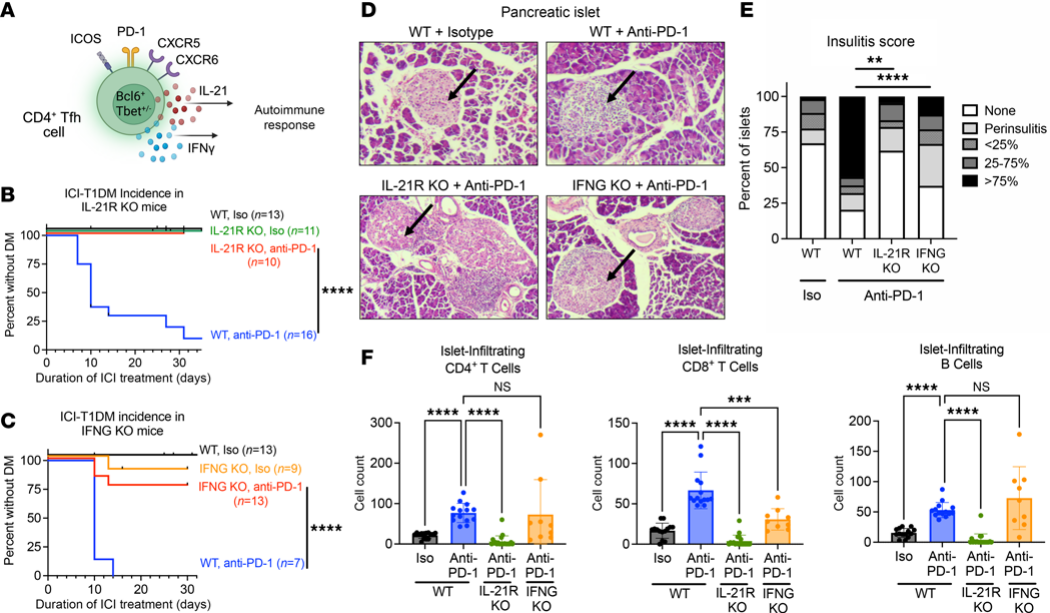
IL-21 and IFN-γ are key cytokine mediators of ICI-T1DM. (**A**) Schematic of cytokine production by Tfh cells. (**B**) Incidence curve for ICI-T1DM in anti–PD-1 treated NOD WT and NOD.*Il21r*^–/–^ (IL-21R KO) mice. WT, Iso (6 males, 7 females); IL-21R–KO, Iso (11 males); IL-21R–KO, anti–PD-1 (8 males, 2 females). (**C**) Incidence curve for ICI-T1DM in ICI-treated NOD WT and NOD.IFN-γ*^–/–^* (IFN-γ–KO) mice during anti–PD-1 treatment. WT, Iso (6 males, 7 females); IFN-γ–KO, Iso (6 males, 3 females); IFN-γ–KO, anti–PD-1 (6 males, 7 females), WT, anti–PD-1 (3 males, 4 females). (**D**) Representative H&E-stained pancreas histology sections of Iso- or anti–PD-1–treated WT, IL-21R–KO, or IFN-γ–KO mice (original magnification, 100×). Arrow indicates an islet of Langerhans. (**E**) Insulitis index determined by histologic analyses of pancreas islet histology across indicated treatment conditions. WT, Iso (5 males, 10 females); WT, anti–PD-1 (6 males, 10 females); IL-21R–KO, anti–PD-1 (4 males, 1 female); IFN-γ–KO, anti–PD-1 (5 males, 5 females). (**F**) Quantification of CD4^+^ T, CD8^+^ T, and B cells from anti–PD-1–treated WT (2 males, 2 females), IL-21R–KO (2 males, 1 female), and IFN-γ–KO (5 males, 4 females) mice or Iso WT (1 male, 3 females), via multi-immunofluorescence staining. Comparisons by log-rank test (**B** and **C**), Fisher’s exact test (**E**), or Brown-Forsythe ANOVA with Welch’s pairwise comparison test (**F**). **P < 0.01, ***P < 0.001, ****P < 0.0001.

We hypothesized that inhibition of Tfh cytokines, specifically IL-21 and IFN-γ (Figure 2A), could attenuate autoimmune attack on the pancreas during anti–PD-1 therapy. IL-21 is a pleiotropic cytokine that can promote effector functions in CD8^+^ T cells (10, 20, 21) and B cell antibody production (43). In humans and mice, CD4^+^ Tfh cells are the primary source of IL-21 (18, 44). Indeed, NOD mice with genetic deletion of IL-21 signaling (NOD.*Il21r*^–/–^, IL-21R KO) were protected from the development of ICI-T1DM during ICI treatment (Figure 2B; *P* < 0.0001 for anti–PD-1 therapy in WT versus IL-21R–KO mice). It is recognized that IL-21 is required for the development of spontaneous T1DM in NOD mice (40, 45), and these data establish a role for IL-21 in ICI-T1DM as well.

IFN-γ is expressed more broadly, including by both CD4^+^ and CD8^+^ T cells in ICI-T1DM (29–31). NOD mice with genetic deletion of the IFN-γ gene (NOD.IFN-γ^–/–^, IFN-γ KO) showed significantly delayed onset of ICI-T1DM (Figure 2C; *P* < 0.0001 for anti–PD-1 therapy in WT versus IFN-γ KO mice). These data confirm a previous nonsignificant trend reported by Perdigoto et al. (31) and are consistent with IFN-γ as a mediator of ICI-T1DM (29). Pancreas histology and insulitis scoring of both anti–PD-1–treated IL-21R–KO and IFN-γ–KO mice confirmed reduced frequency of heavily infiltrated islets (>75%) compared with anti–PD-1–treated WT mice (Figure 2, D and E). With decreased incidence of diabetes and severity insulitis, we wondered how loss of these cytokine signals changed the immune infiltrate composition within islets (Figure 2F). Loss of IL-21 signaling showed a marked decrease in islet-infiltrating CD8^+^ T, CD4^+^ T, and B cells, consistent with the role of IL-21 in recruiting immune cells to tertiary lymphoid structures. By contrast, loss of IFN-γ resulted in fewer islet-infiltrating CD8^+^ T cells, but there was no difference in CD4^+^ T or B cell accumulation, suggesting IFN-γ protection acts downstream from immune cell accumulation in ICI-T1DM. In summary, our data identify a role for IL-21^+^IFN-γ^+^ Tfh cells in ICI-T1DM and demonstrate that inhibition of these 2 cytokine pathways can prevent the development of autoimmunity.

### JAK1/2 inhibition via ruxolitinib prevents ICI-induced DM in NOD mice.

**Figure 3:**
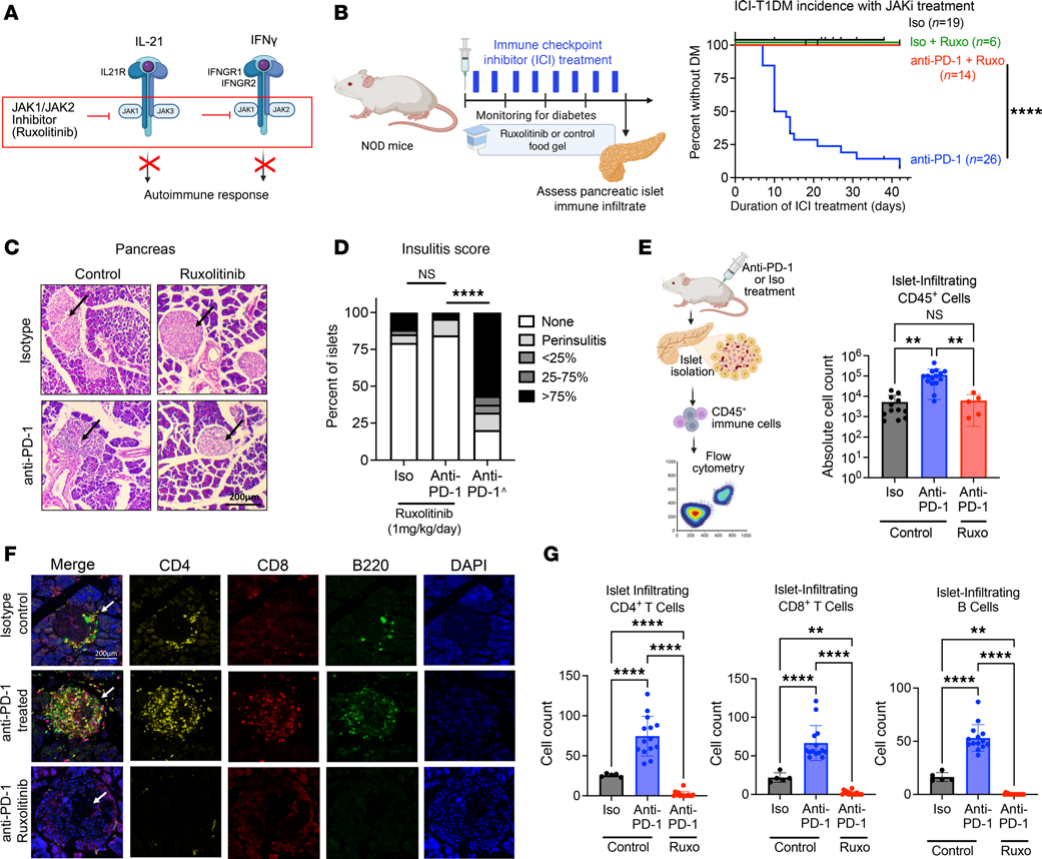
JAKi ruxolitinib provides robust protection against ICI autoimmune DM. (**A**) Proposed JAK signaling inhibition downstream from IL-21 and IFN-γ to halt autoimmune response. (**B**) Schematic for treatment of mice with JAKi ruxolitinib (left) and incidence of autoimmune DM (right) in NOD mice treated with anti–PD-1 immunotherapy or Iso, and ruxolitinib or control food gel. Iso (6 males, 13 females); Iso + Ruxo (3 males, 3 females); anti–PD-1 + Ruxo (6 males, 8 females); anti–PD-1 (8 males, 18 females). (**C**) Representative H&E-stained pancreas histology sections of anti–PD-1 or Iso-treated NOD mice (original magnification, 100×) fed ruxolitinib or control food. Arrow indicates an islet of Langerhans. (**D**) Insulitis index of anti–PD-1 or Iso-treated NOD mice given ruxolitinib (Iso: 1 males, 1 females; anti–PD-1: 4 males, 4 females); ^data for anti–PD-1 mice given control chow are the same as shown in Figure 2E, reshown here for comparative purposes. (**E**) Schematic and absolute cell counts of pancreatic islet–infiltrating CD45^+^ cells, as determined by flow cytometry, across Iso + vehicle (*n* = 12), anti–PD-1 + vehicle (*n* = 15), and anti–PD-1 + ruxolitinib (*n* = 5) conditions. Each point represents data from 1 animal. (**F**) Representative multi-immunofluorescence staining and microscopy images (original magnification, 40×) of CD4, CD8, B220, and DAPI in the islet of Langerhans across experimental conditions. Arrow indicates islet in merge images. (**G**) Quantification of CD4^+^ T cell, CD8^+^ T cell, and B220^+^ B cell counts per pancreatic islet of indicated treatment condition by immunofluorescence from mice treated with Ruxo + anti–PD-1 (3 males, 2 females); data for Iso or anti–PD-1 treated mice given control chow are the same as shown in Figure 2F, reshown here for comparative purposes. Data are presented as mean ± SD (**E** and **G**). Comparisons by log-rank test (**B**), Fisher’s exact test (**D**), or ANOVA with Welch’s correction and pairwise comparison by Tukey’s test (**E** and **G**). **P < 0.01, ****P < 0.0001.

With the expanding use of ICI therapies and the rising number of patients affected by IrAEs, there is a pressing clinical need for near-term strategies to prevent or reverse treatment-associated autoimmunity. To this end, we wondered whether JAKi, a group of clinically approved agents used in spontaneous autoimmune diseases (46–49), could prevent ICI-T1DM. JAK signaling is central to many T cell immune responses, including downstream signals of IL-21 (43) and IFN-γ (50) (JAK1/2 and JAK1/3, respectively) (Figure 3A). Using our mouse model, we tested whether treatment with the JAK1/2 inhibitor ruxolitinib could delay development of ICI-T1DM (Figure 3B). Notably, while anti–PD-1–treated mice on control food rapidly developed autoimmune diabetes, ruxolitinib therapy prevented ICI-associated autoimmunity, with no mice developing overt DM (Figure 3B; *P* < 0.0001).

Histologic analysis of pancreatic islets from anti–PD-1–treated mice given ruxolitinib showed minimal immune infiltrate (Figure 3C), with insulitis scores comparable with isotype-treated controls (Figure 3D). We confirmed reduced immune infiltrates in ruxolitinib-fed mice using flow cytometry analysis of immune cells in isolated pancreatic islets. Compared with anti–PD-1–treated mice, those additionally given ruxolitinib had significantly reduced islet-infiltrating CD45^+^ immune cells (*P* < 0.01), comparable with isotype-treated controls (Figure 3E). Our further characterization and quantification of immune infiltrates in pancreatic islet infiltrates using multiparameter immunofluorescence staining of tissue specimens (Figure 3F) demonstrated that CD4^+^ T cells, CD8^+^ T cells, and B220^+^ B cells accumulated within the islets of anti–PD-1–treated mice and were significantly decreased by the addition of ruxolitinib therapy (Figure 3G; *P* < 0.0001 for all cell types).

In addition, we tested whether ruxolitinib could reverse ICI-T1DM. This is clinically relevant because the development of ICI-T1DM in patients is usually detected after clinical diabetes development. Here, groups of anti–PD-1–treated mice were randomized to treatment with ruxolitinib or vehicle control after the development of diabetes (blood glucose > 200 mg/dL) (Supplemental Figure 3A). Ruxolitinib-treated mice had improved glycemic control compared with vehicle controls (Supplemental Figure 3B; *P* < 0.0005, and Supplemental 3C) and had decreased insulitis scores on pancreas histology (Supplemental Figure 3, D and E). We also tested the duration of JAKi protection by treating a cohort of mice with 1 week of ruxolitinib followed by control chow during anti–PD-1 treatment. Compared with mice given no ruxolitinib, those with transient ruxolitinib treatment had delayed onset of ICI-T1DM with anti–PD-1 therapy (median onset 22 days compared with 9.5 days; Supplemental Figure 3F), but protection was transient, suggesting that protection aligns with active therapy and 2–3 days of residual drug effects. Finally, prior studies have shown PD-1 blockade to be the primary driver of accelerated autoimmunity in adult mice (51–53), but ICI-T1DM can also develop in patients with cancer who are treated with combination ICI regimens (e.g., anti–PD-1 + anti-cytotoxic T lymphocyte antigen [CTLA-4]) (Supplemental Figure 4A) (4–6). As in mice that received anti–PD-1 monotherapy, JAKi treatment prevented the development of ICI-T1DM in mice treated with combination anti–PD-1 + anti–CTLA-4 (Supplemental Figure 4B). In summary, these data show potent in vivo protection against ICI-T1DM using a clinically available JAKi.

### JAKi therapy disrupts the CD4^+^ Tfh cell compartment to prevent ICI-T1DM.

**Figure 4:**
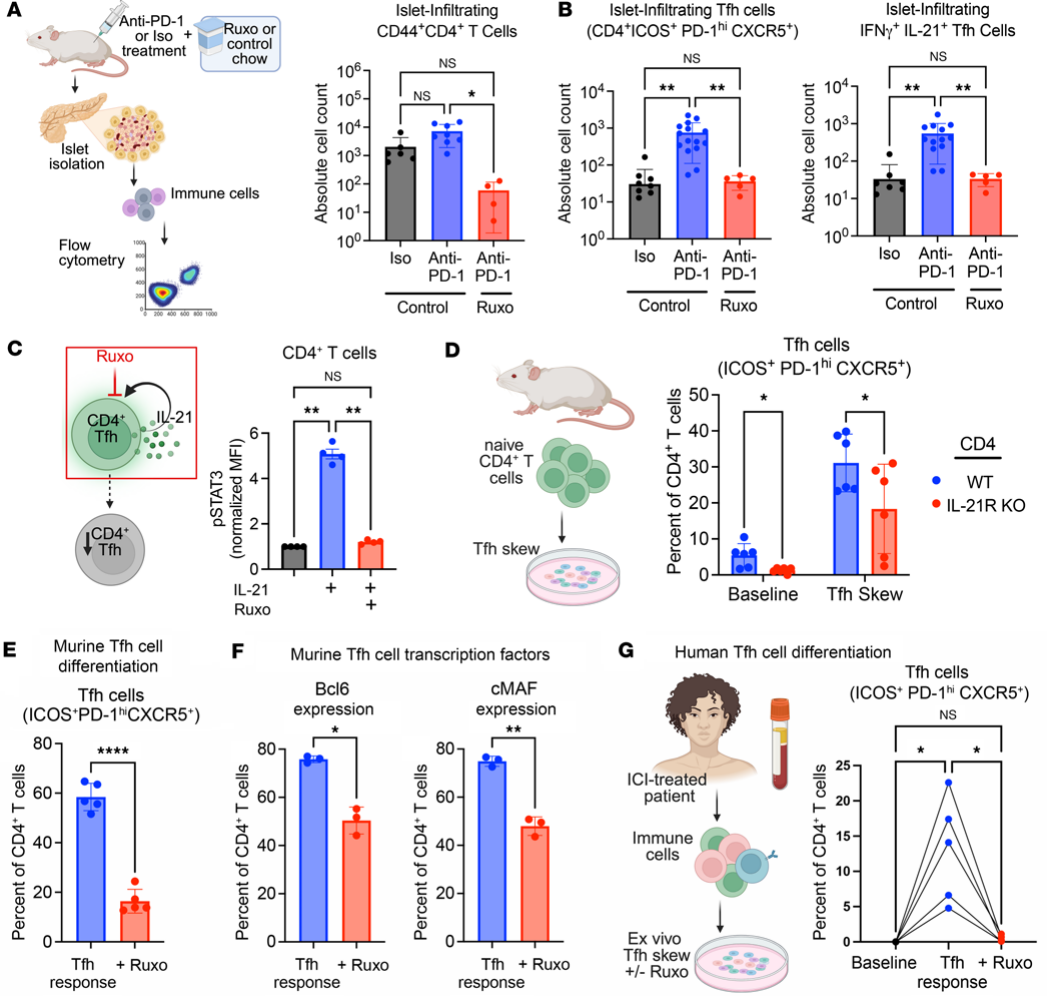
JAKi treatment reduces CD4^+^ Tfh cell response in mice and humans. (**A**) Schematic and quantification of islet-infiltrating, CD44^+^CD4^+^ T cells among mice treated with Iso + vehicle (*n* = 6), anti–PD-1 + vehicle (*n* = 8), and anti–PD-1 + ruxolitinib (*n* = 4), via flow cytometry analysis. (**B**) Quantification of islet-infiltrating, ICOS^+^PD-1^hi^CXCR5^+^CD4^+^ Tfh (left) and IL-21^+^IFN-γ^+^ Tfh cells (right), among mice treated with Iso + vehicle (*n* = 9–12), anti–PD-1 + vehicle (*n* = 13–15), and anti–PD-1 + ruxolitinib (*n* = 5), via flow cytometry. (**C**) Schematic of proposed action of JAKi on CD4^+^ Tfh cells through blockade of autocrine IL-21 signaling (left). STAT3 phosphorylation in murine CD4^+^ T cells in response to IL-21 (100 ng/mL), ruxolitinib (10 μM), or vehicle in vitro, assessed by flow cytometry (right). (**D**) Effect of IL-21 receptor genetic deletion in CD4^+^ T cells on Tfh cell induction in vitro, following a 3-day Tfh skew of naive CD4^+^ T cells with anti–PD-1. (**E**) Frequency of CD4^+^ T cells expressing a Tfh cell phenotype (CD4^+^ICOS^+^PD-1^hi^CXCR5^+^) following a 3-day Tfh skew of naive CD4^+^ T cells in the presence of ruxolitinib (10 μM) or vehicle in vitro, assessed by flow cytometric analysis. (**F**) Expression of canonical Tfh transcription factors Bcl6 and cMAF in murine CD4^+^ T cells following a 3-day Tfh skew in the presence ruxolitinib (Ruxo, 10 μM) or vehicle. (**G**) Comparison of Tfh cell response in PBMC specimens from ICI-treated individuals at baseline and following a 3-day Tfh skew with JAKi ruxolitinib (Ruxo, 10 μM) or vehicle, measured by flow cytometry. Each point represents data from one replicate (**C**–**F**); experiments repeated at least twice or animal (**A** and **B**), and data are presented as mean ± SD. For human studies (**G**), connected points represent data from 1 individual. Comparisons by Brown-Forsythe and Welch ANOVA (**A** and **B**), 1-way ANOVA (**C** and **D**) with subsequent pairwise comparisons, Welch’s *t* test (**E** and **F**), or 1-way ANOVA for paired samples (**G**). *P < 0.05; **P < 0.01, ****P < 0.0001.

JAKi treatment in our mouse model of ICI-T1DM led to significantly fewer effector CD44^+^CD4^+^ T cells (Figure 4A; *P* < 0.05) and Tfh cells (ICOS^+^PD-1^hi^CXCR5^+^) within pancreatic islets (Figure 4B; *P* < 0.01). IL-21 signaling to CD4^+^ T cells supports Tfh cell differentiation and relies upon JAK signaling. Therefore, we predicted that JAKi therapy may attenuate Tfh cell responses by preventing autocrine IL-21 signaling in CD4^+^ T cells. Indeed, in vitro treatment of CD4^+^ T cells with ruxolitinib prevented JAK-mediated intracellular STAT3 phosphorylation in response to IL-21 stimulation (Figure 4C; *P* < 0.01). In addition, CD4^+^ T cells with genetic IL-21 receptor loss (IL-21R KO) had reduced differentiation to Tfh cells in vitro (Figure 4D; *P* < 0.05). These data show that, in addition to blocking the downstream effects of Tfh cell cytokines (i.e., IL-21 and IFN-γ), JAKi therapy is associated with decreased Tfh cells in vivo.

To better understand the effect of JAKi on Tfh cell expansion in ICI-treated mice, we evaluated naive CD4^+^ T cells under Tfh skewing conditions (54) with and without ruxolitinib in vitro. Flow cytometry analysis revealed that ruxolitinib reduced markers of Tfh cell differentiation in CD4^+^ T cells (Figure 4E; *P* < 0.0001) and downregulated the expression of Bcl6 (*P* < 0.05) and cMAF (*P* < 0.01), a transcription factor required for IL-21 expression in Tfh cells (Figure 4F).

We then evaluated whether JAKi treatment similarly impaired Tfh cell responses in humans. Using peripheral blood specimens from patients treated with ICI therapy, we compared the frequency of CD4^+^ Tfh cells after culture under Tfh-skewing conditions ex vivo. Indeed, Tfh cell differentiation was significantly decreased by JAKi ruxolitinib (Figure 4G**,** P < 0.05). Thus, JAKi decreased Tfh cell induction in murine and human CD4^+^ T cells, suggesting a mechanism by which the development of ICI-T1DM can be prevented in vivo. Taken together, these data support JAKi as a potential near-term therapeutic strategy by which we can target CD4^+^ Tfh cell responses in patients with ICI-T1DM.

## Discussion

The benefits of ICI therapy hold great promise for patients with many types of cancer, but their use is limited by IrAEs. Among the most severe IrAEs is ICI-T1DM, which leads to destruction of pancreatic islets and life-long insulin dependence. In addition, because of the rapid progression of islet loss compared with spontaneous autoimmune T1DM, patients with ICI-T1DM more frequently present with diabetic ketoacidosis (nearly 80%–90%) at diagnosis and require hospital admission to an intensive care unit (6, 55).

In this study, we provide evidence for robust protection of ICI-T1DM with ruxolitinib, an FDA-approved and clinically available JAK1/2 inhibitor. This builds upon a prior report by Ge at al. (56) evaluating a preclinical selective JAK1 inhibitor for ICI-T1DM in mice and the recent successful phase 2 trial of JAKi baricitinib in spontaneous T1DM (16). Furthermore, studies combining JAKi and anti–PD-1 therapy in patients with non-small cell lung cancer or Hodgkin’s lymphoma as well as in a preclinical model of breast cancer reported improved cancer outcomes (57–59). Thus, JAKi therapies may be a feasible and near-term approach to reducing toxicity from severe IrAEs such as ICI-T1DM.

On the other hand, JAK signaling is important for many T cell immune responses and can induce broad immune suppression when used against spontaneous autoimmune diseases. It is possible, therefore, that JAKi treatment may also impair desired immune responses during cancer immunotherapy. Thus, an aim of our present study was to delineate the cellular mechanisms underlying immune protection during JAKi therapy so that more targeted immunosuppressive strategies could be developed. Prior studies have shown that effector IFN-γ^+^CD8^+^ T cells contribute to autoimmune attack on pancreatic β-islet cells during ICI therapy (29–31). Given the importance of IFN-γ and CD8^+^ T cells to ICI anti-tumor immune responses (35), we focused on the less explored role of CD4^+^ T cells with the aim of identifying driving immune mechanisms that could be targeted in patients with cancer to reduce IrAEs while preserving efficacy.

Indeed, we found a significant contribution from CD4^+^ T cells in the immunopathogenesis of ICI-T1DM. Specifically, our findings support a critical role for CD4^+^ Tfh cells in the immunopathogenesis of ICI-T1DM. Within islet immune infiltrates, we demonstrated antigen-specific multifunctional IL-21^+^IFN-γ^+^CD4^+^ Tfh cells that were enriched in ICI-treated mice with diabetes. Wherry and colleagues also showed expansion of circulating CD4^+^ Tfh cells in anti–PD-1–treated patients after influenza vaccination correlated with the development of IrAEs (*P* = 0.06) (22). Expanding upon this role in the periphery, we showed increased Tfh cells in the thyroid tissue of patients with ICI-thyroiditis (10) and now extend their role to ICI-T1DM. Our data also highlight Tfh cells as a source of 2 important cytokines in the autoimmune response, namely IFN-γ and IL-21. Hu and colleagues previously showed the IFN-γ contributes to immune cell migration into pancreas islets and macrophage activation in ICI-T1DM (29). While a role for IL-21 in ICI-T1DM has not been described, we showed in mice and humans with ICI-thyroiditis that IL-21 from CD4^+^ cells could augment effector molecules (IFN-γ, granzyme B) and chemokine receptors on thyrotoxic CD8^+^ T cells (10). In addition, IL-21 is well known to promote spontaneous T1DM (45).

To further explore how JAKi may modulate this pathogenic CD4^+^ T cell subset, we evaluated Tfh cells in both ICI-treated mice and ex vivo using human specimens. In both contexts, we observed decreased Tfh cell frequency. While JAKi are known to block the downstream effects of multiple T cell cytokines (43, 46), we showed that they can also block Tfh cell differentiation. These dual mechanisms may be collectively responsible for the benefit of JAKi treatment seen in spontaneous and cancer immunotherapy–associated autoimmune diseases. Furthermore, our studies revealed a vulnerability in autocrine IL-21 CD4^+^ T cell signaling as a potential targeted approach to reduce ICI-associated autoimmunity. Future studies are warranted to further evaluate JAK inhibition and the IL-21 CD4^+^ Tfh cell axis as potential therapeutic targets for reversal of severe IrAEs in patients treated for ICI, as well as the effect on ICI antitumor immune responses. In conclusion, our studies not only indicate strong preclinical application of JAK1/2 inhibition in the protection of ICI-T1DM development but demonstrate a critical role for IL-21^+^IFNγ^+^CD4^+^ Tfh cells in driving the mechanism of autoimmune attack and pancreatic injury in ICI-T1DM.

## Methods

### Sex as a biologic variable.

IrAEs occur in both males and females. Therefore, for animal studies, both male and female mice were used in equal proportions. For human studies, both male and female individuals were eligible for participation and included.

### Antibodies and reagents.

Primary immune cells were cultured in RPMI-1640 complete media (supplemented with 10% FBS, 2 mM L-glutamine, 1 mM HEPES, nonessential amino acids, and antibiotics (penicillin and streptomycin), with 50 μM β-mercaptoethanol (2ME) (all reagents from Thermo Fisher Scientific). ICI antibodies used were anti-mouse PD-1 (clone RPM1-14, BE0146), CTLA-4 (clone 9D9, BE0164), and isotype control (clone 2A3 BE0089) (all from Bio X Cell). Antibodies were diluted in sterile PBS for use. Ruxolitinib was obtained from MCE and diluted in sterile DMSO (MilliporeSigma) for in vitro use. For animal experiments, ruxolitinib was prepared at 1 g/kg in Nutra-Gel Diet (Bio-Serv, F5769-KIT) chow as previously described (60).

### Mouse studies.

Animal studies were approved by the UCLA Animal Research Committee (protocol nos. C21-039 and C24-012). NOD/ShiLtJ (NOD, 001976), NOD.*Il21r*^–/–^ (IL-21R KO, 034163), NOD.*Trca*^–/–^ (TCRα KO, 004444), NOD.IFN-γ*^–/–^* (IFN-γ KO, 002575), and NOD/SCID (001303) mice were obtained from The Jackson Laboratory. Male and female mice were used in equal proportions. Mice were used at 7–9 weeks of age unless otherwise noted. Mice were housed in a specific pathogen–free barrier facility at UCLA. Mice in different experimental groups were cohoused.

### ICI treatment of mice.

Mice were randomized to continuous twice-weekly treatment with anti–mouse PD-1 (clone RPM1-14) and/or CTLA-4 (clone 9D9) or isotype control antibody (clone 2A3, Bio X Cell), at 10 mg/kg/dose i.p., as described previously (24). During treatment, mice were monitored daily for activity and appearance and monitored twice weekly for weight and glucosuria. Mice developing glucosuria or blood glucose > 200 mg/dL were treated with 10 units of s.c. NPH insulin daily. At the end of ICI treatment course, mice were euthanized and perfused with 10 mL of sterile phosphate-buffered saline (PBS) by intracardiac puncture, and fresh tissues were immediately collected for histology or dissociated for analysis of immune infiltrates by flow cytometry. Predetermined endpoints for early euthanasia included > 20% weight loss and glucosuria not resolved by insulin therapy, as per IACUC protocols.

For the evaluation of immune infiltrates by flow, islet-infiltrating lymphocytes were collected following an isolation protocol described by Villarreal et al. (61). Fresh pancreas specimens were perfused with 3 mL of a collagenase P solution (1 mg/mL Collagenase P in HBSS, supplemented with 0.05% BSA) via the ampulla of Vater, dissected away from surrounding tissue, and mechanically digested in a 37°C thermo-shaker at 100–120 rpm for 13 minutes. Pancreatic islets were then purified using a Histopaque-1077 density gradient (Sigma-Aldrich).

Spleen cells were isolated by mechanical dissociation and passage through a 40 μm filter.

### Ruxolitinib treatment of mice.

For animal experiments, ruxolitinib was prepared at 1 g/kg in Nutra-Gel Diet (Bio-Serv, F5769-KIT) chow as previously described (60). Nutra-Gel Diet chow without ruxolitinib served as control food. For DM reversal experiments, mice were treated with anti–PD-1 twice weekly and monitored daily for blood glucose levels by tail prick. Mice with hyperglycemia (blood glucose level > 200 mg/dL), were randomized into either ruxolitinib or control chow groups, and ICI treatment was stopped. Ruxolitinib therapy was given as a single oral gavage dose of 1.25 mg on day of hyperglycemia onset, followed by ruxolitinib chow (1 g/kg) as above. All diabetic mice were assessed daily for blood glucose and treated with NPH insulin if hyperglycemic (10 units for blood glucose > 300 mg/dL, 5 units for blood glucose between 200 and 300 mg/dL). Mice with persistent hyperglycemia not resolved by insulin therapy after 4 days were euthanized per IACUC protocol.

### In vitro assessment of primary murine immune cells.

Splenocytes were isolated from healthy NOD.WT mice by mechanical dissociation. Naive CD4^+^ T and CD8^+^ T cells were isolated by magnetic bead separation as above and cultured at 5 × 10^5^ cells/well in 12-well plates in complete media with 2ME. For Tfh skew, cells were stimulated with plate-bound anti–murine CD3 (Invitrogen, clone 145-2C11; 1 μg/mL) and soluble anti–murine CD28 (Invitrogen, clone 37.51; 1 μg/mL), anti–murine IFN-γ (Bio X Cell XMG1.2,10 μg/mL), anti–murine IL-4 antibodies (BD Biosciences, catalog 554385; 10 μg/mL), anti–murine TGF-β (Thermo Fisher Scientific, catalog 16-9243-85; 20 μg/mL), recombinant mouse IL-6 (PeproTech; 10 ng/mL), and IL-21 (PeproTech; 10 ng/mL), as previously described (54). Cells were evaluated on day 3 by flow cytometry. Experiments were repeated at least twice.

### Histology and immunofluorescence.

Harvested tissues were fixed in Zinc for at least 48 hours and then stored in 70% ethanol. Organs were embedded in paraffin, sectioned (4µ), and stained with H&E by the UCLA Translational Pathology Core Laboratory. Insulitis was quantified by blinded assessment of H&E sections as previously reported (62). Images were acquired on an Olympus BX50 microscope using Olympus CellScans Standard software. Images were brightened uniformly for publication in Photoshop.

### Antibody clones and dilutions.

Appropriate positive and negative controls were used for all stains. DAPI, Opal 520 stain for B Cells, Opal 570 stain for CD4^+^ T cells, and Opal 690 stain for CD8^+^ T cells were used. Images of the islet were exported to Photoshop and then analyzed in ImageJ (NIH) for total islet area and quantification of each cell type.

### Patients.

Peripheral blood specimens from patients with cancer treated with ICI therapy were collected from patients treated in endocrinology and oncology clinics at UCLA or UCSF under IRB-approved protocols. Patients were divided for development of IrAEs, including ICI-T1DM, during ICI therapy and those with no history of IrAEs. ICI-T1DM was defined by new-onset hyperglycemia with low c-peptide and insulin dependence during ICI cancer therapy, consistent with current guidelines from the National Comprehensive Cancer Network (NCCN) Management of Immunotherapy Toxicities guidelines (7). Other IrAEs were classified based upon NCCN and Common Terminology Criteria for Adverse Events (CTCAE v5) criteria. Individuals defined as “no IrAE” had no evidence of any grade ≥ 2 IrAE or preexisting autoimmune disease. Individual data are presented in Supplemental Table 1.

### In vitro assessment of primary human immune cells.

Peripheral blood mononuclear cells were isolated from whole blood by density gradient centrifugation using Ficoll-Paque (Cytiva). For Tfh skew, cells were stimulated with plate bound anti–human CD3 (Bio X Cell, clone UCHT1; 1 μg/mL) and soluble anti–human CD28 (Bio X Cell, clone 9.3; 1 μg/mL), recombinant human IL-12 (PeproTech; 10 ng/mL), and Activin A (R&D Systems; 100 ng/mL), as previously described (23). Cells were evaluated on day 3 by flow cytometry. Experiments were repeated at least twice.

### Flow cytometry.

For staining, single-cell suspensions were resuspended in FACS buffer consisting of 0.5 mM EDTA, and 2% FBS in PBS at 1 × 10^6^ cells/mL. Cells were stained in LIVE/DEAD Fixable Yellow Dead Cell Stain (Thermo Fisher Scientific) for 30 minutes prior to surface staining. Cells were then stained with fluorescence-conjugated antibodies as indicated in Supplemental Table 2. For intracellular staining, after surface staining, cells were fixed and permeabilized using cytoplasmic fixation and permeabilization kit (BD Biosciences), per manufacturer instructions, with a 20-minute fixation step at 4°C. To assess intracellular cytokines, cells were incubated in complete RPMI-1640 media with 50 μM 2ME for 4 hours with ionomycin (100 ng/mL) (Thermo Fisher Scientific) and PMA (50ng/mL) (MilliporeSigma) in the presence of Brefeldin A (BioLegend) before staining. For intranuclear staining of phosphorylated signaling proteins, cells were fixed and permeabilized with 4% paraformaldehyde (PFA) for 15 minutes at room temperature, and ice-cold 100% methanol at 4°C for 45 minutes. For intranuclear staining of transcription factors, cells were fixed and permeabilized using a transcription factor fixation and permeabilization buffer kit (Thermo Fisher Scientific), following the provided manufacturer protocol including a 30-minute fixation at room temperature. For tetramer staining, BDC2.5-mimotope tetramer (CD4^+^ T cell I-Ag7, BDC2.5 mimotope [peptide sequence AHHPIWARMDA]) fluorescently conjugated reagents were obtained from the NIH Tetramer Core and stained at room temperature for 30 minutes as previously described (63). After staining, cells were washed twice in FACS buffer and analyzed by flow cytometry on an Attune NxT 6 cytometer (Thermo Fisher Scientific).

Cell counts are shown as the relative frequency of live, gated single cells unless otherwise noted. For the determination of infiltrating cells within pancreatic islets, absolute cell counts were determined using counting beads (Thermo Fisher Scientific, C36995), following the manufacturer’s protocol. Beads were added to pancreatic islet samples at a concentration of 1 μL/7 μL of sample volume. Representative gating strategies are shown in Figure 1 and Supplemental Figures 1 and 2.

### Statistics.

Statistical analyses were performed using GraphPad Prism software (v10). Comparisons among multiple groups for continuous data were made using ANOVA (paired sample analysis when appropriate) or ANOVA with Welch correction with no assumption for equal variances, with subsequent pairwise comparisons by Tukey’s or Dunnett’s tests. Figure legends specify 1-way or 2-way ANOVA. Nonparametric data were evaluated using the Mann-Whitney *U* test. Comparisons between 2 groups were done by 2-sided Student’s *t* test with Welch correction with no assumption for equal variances. Differences in diabetes incidence over time were compared using Log Rank test. When multiple comparisons were performed, adjusted *P* values were shown. A *P* value less than 0.05 was considered significant.

### Study approval.

All animal experiments were conducted under UCLA IACUC-approved protocols (nos. 2024-012 and 21-039) and complied with the Animal Welfare Act and the *Guide for the Care and Use of Laboratory Animals* (National Academies Press, 2011). All human experiments were conducted under UCLA (no. 23-000015) and UCSF (no. 10-02467) IRB-approved protocols, and participants provided written informed consent.

### Data availability.

Values for all data points in graphs are reported in the Supporting Data Values file. Additional data available upon reasonable request.

## Author contributions

NLH, JGO, K Kimbrell, JL, LNS, and MGL designed the study. NLH, JGO, K Kimbrell, JL, LNS, EYK, EMP, SJW, K Kim, JO, and JNN conducted experiments and acquired data. NLH, JGO, K Kimbrell, JL, LNS, MAS, and MGL analyzed and interpreted the data. TEA, ZQ, and MGL designed the clinical studies, enrolled patients, and provided clinical specimens. NLH and JGO wrote the manuscript. All authors reviewed and edited the manuscript. NLH and JGO contributed equally to this work and are listed alphabetically by surname.

## Supplementary Material

Supplemental data

Supporting data values

## Version 1 07/08/2025

Electronic publication
