# Supplementary material for: Polyfunctional T follicular helper cells drive checkpoint-inhibitor diabetes and are targeted by JAK inhibitor therapy
Source: JCI Insight. 2025 Jul 8;10(13):e188843. doi: 10.1172/jci.insight.188843 (PMC12288894; doi:10.1172/jci.insight.188843)
Supplement: Supplemental data [file jciinsight-10-188843-s180.pdf]

Supplemental Tables

	Age (years) at start of ICI	Sex	Immunotherapy	Cancer type	IrAEs	Treatment prior to immunotherapy	Leukocyte count at time of sample acquisition	C-peptide (ng/mL), serum glucose (mg/dL) at diagnosis	Autoantibodies
Individuals with No IrAEs (Fig. 1)	47	Male	Pembrolizumab	Metastatic Melanoma	No significant IrAE	Vemurafenib	WBC 11, ALC 1.88	NA	NA
	43	Female	Pembrolizumab	Head and Neck SCC	No significant IrAE	Surgery, Carboplatin/Paclitaxel; Docetaxel, Cisplatin and Fluorouracil, Radiation	WBC 7.4, ALC 1.59	NA	NA
	47	Female	Pembrolizumab	Diffuse type Gastric Carcinoma	No significant IrAE	Epirubicin, Oxaliplatin, Capecitabine; Surgery; Carboplatin/Paclitaxel	WBC 5.8, ALC 1.74	NA	NA
	55	Male	Pembrolizumab	Head and Neck SCC	No significant IrAE	Surgery, Cisplatin,	WBC 5.1, ALC 1.27	NA	NA
	75	Male	Atezolizumab	Pancreatic Cancer	No significant IrAE	Gemcitabine, Nab-Paclitaxel; Atezolizumab	WBC 7.3, ALC 1.99	NA	NA

	Age (years) at IrAE	Sex	Immunotherapy	Cancer type	IrAEs	Treatment prior to immunotherapy	Leukocyte count at time of sample acquisition	C-peptide (ng/mL), serum glucose (mg/dL) at diagnosis	Autoantibodies
Individuals with ICI-T1DM (Fig. 1)	64	Male	Nivolumab	Metastatic Melanoma	**ICI-T1DM**	Surgery	WBC 5.4, ALC 1.05	<0.1, 224	Negative
	53	Male	Pembrolizumab	Metastatic Melanoma	ICI-Hypophysitis, ICI-Thyroiditis, **ICI-T1DM**, Hepatitis, Vitiligo	Surgery	WBC 5.5, ALC 1.86	1.5, 424	Negative
	60	Male	Nivolumab	Metastatic Melanoma	ICI-Colitis, **ICI-T1DM**	Ipilimumab/Nivolumab, Radiation	WBC 7.3, ALC 1.09	0.8, 506	Negative
	56	Female	Nivolumab	Mucosal Melanoma of Vagina	ICI-Pneumonitis, **ICI-T1DM,** ICI-Colitis	Radiation		NA	NA
	59	Female	Nivolumab	Melanoma	ICI-thyroiditis, **ICI-T1DM,** ICI-Sjogrens	None	WBC 5.6, ALC 0.84	0.4, 336	Negative

	Age (years) at IrAE	Sex	Immunotherapy	Cancer Type	IrAEs	Treatment prior to immunotherapy	Leukocyte count at time of sample acquisition	C-peptide (ng/mL), serum glucose (mg/dL) at diagnosis	Autoantibodies
ICI-treated patients (Fig. 5)	65	Male	Nivolumab	Metastatic Melanoma	**ICI-T1DM,** ICI-Thyroiditis	None	WBC 7.0, ALC 1.01	<0.5, 170	Negative
	69	Male	Pembrolizumab	Metastatic Melanoma	ICI-Thyroiditis, ICI-Colitis, **ICI-T1DM,** ICI-Arthritis	Ipilimumab/Nivolumab	WBC 4.6, ALC 1.47	<0.5, 105	Positive IA2 Ab
	71	Female	Pembrolizumab	Triple Negative Breast Cancer	ICI-Thyroiditis, ICI-Hypophysitis	Carboplatin, Taxotere with Pembrolizumab	WBC 8.4, ALC 1.48	NA	NA
	34	Female	Nivolumab	Metastatic Melanoma	ICI-Hypophysitis, ICI-Pneumonitis, ICI-Hepatitis	Nivolumab, Relatlimab; Binimetinib, Encorafenib	WBC 7.3, ALC 2.43	NA	NA
	70	Male	Pembrolizumab	Urothelial Cancer	ICI-Psoriasis, ICI-Thyroiditis, ICI-Arthritis, ICI-Colitis	Disitamab Vedotin	WBC 8.9, ALC 1.04	NA	NA

Supplemental Table 1. Demographic and clinical data for patient specimens. ALC, absolute lymphocyte count; BG, blood glucose; DKA, diabetic ketoacidosis; ICI, Immune checkpoint inhibitor; IrAE, Immune related Adverse Event; NA, not applicable or data not available; T1DM, type 1 diabetes mellitus; TPO, thyroid peroxidase; SCC, squamous cell carcinoma; WBC, white blood cell. IrAEs are listed in chronological order of occurrence.

Mouse antibodies

Target	Fluorescence	Clone	Vendor
BCL6	APC-Cy7	K112-91	BD Biosciences
CD4	e450	GK1.5	Invitrogen
CD4	PE	RM4-5	Biolegend
CD44	PerCPCy5.5	IM7	Biolegend
CD44	Brilliant Violet 711	IM7	Biolegend
CD8	APC	53-6.7	Biolegend
cMAF	PE-Cy7	sym0F1	Invitrogen
CXCR5	FITC	L138D7	Biolegend
CXCR6	FITC	SA051D1	Biolegend
ICOS	PerCPCy5.5	C398.4A	Biolegend
IL-21	PE	mhalx21	Invitrogen
IFNy	APC	XMG1.2	Biolegend
pSTAT3	unconjugated	D3A7	Cell Signaling
PD-1	APC	J43	Invitrogen
PD-1	PE-Cy7	J43	Invitrogen
TBET	Brilliant Violet 605	4B10	Biolegend

Human antibodies

Target	Fluorescence	Clone	Vendor
BCL6	APC-Cy7	K112-91	BD Biosciences
CD4	PE	RPA-T4	Biolegend
CXCR5	AF488	J252D4	Biolegend
ICOS	PE-Cy7	ISA-3	Invitrogen
PD-1	e450	MIH4	Invitrogen

Supplemental Table 2. Flow cytometry antibodies.

Supplemental Figures

Supplemental Figure 1

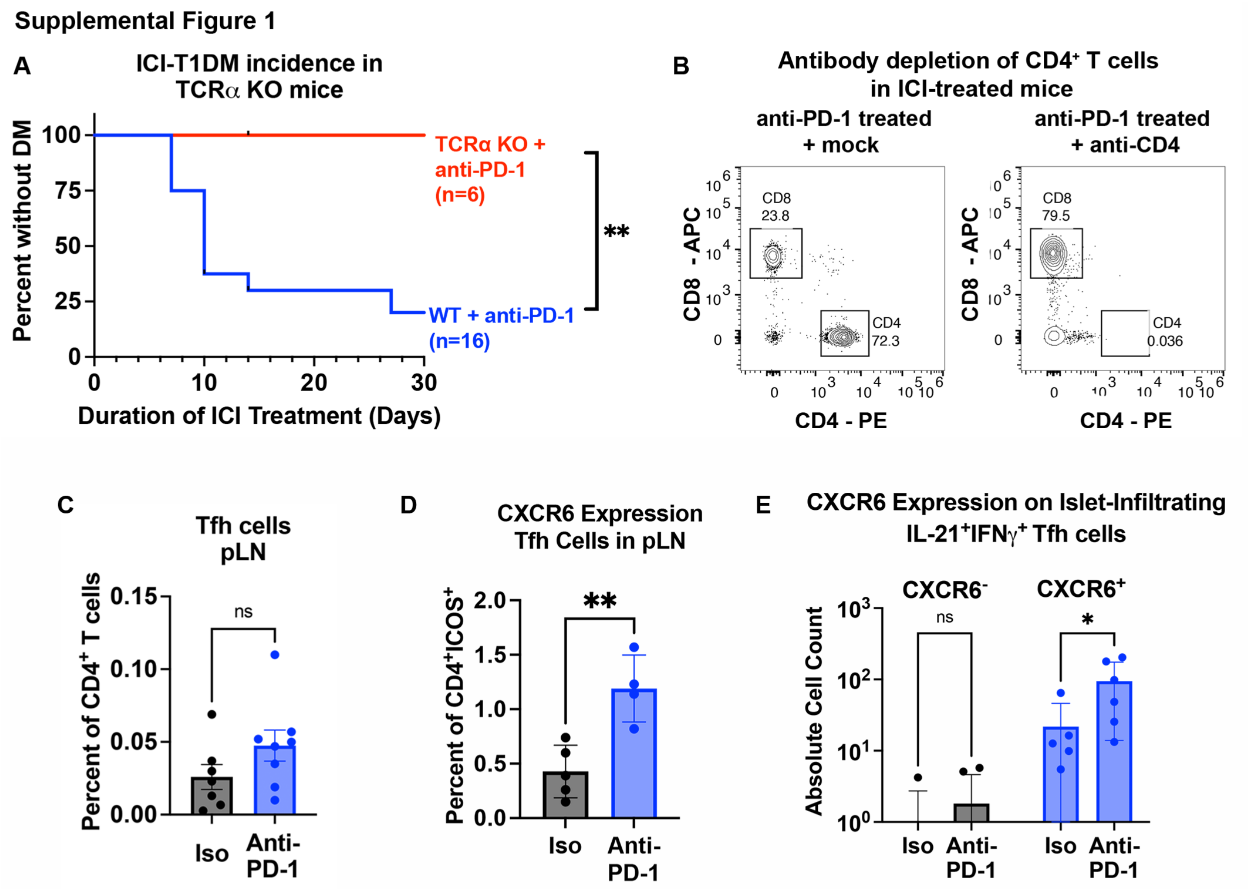

Supplemental Figure 1. CD4^{+} and CD8^{+} T cells are required for the development of immune checkpoint inhibitor autoimmune diabetes in NOD mice.

(A) Incidence of autoimmune diabetes mellitus (DM) in NOD wildtype (WT) (8 males, 8 females) or NOD.*Tcra*$^{-/-}$ (Tcrα KO) mice (3 males, 3 females) treated with anti-PD-1 or isotype control.

(B) Representative flow cytometry plots of splenocytes from NOD mice treated anti-PD1 demonstrating CD4^{+} T cell depletion with anti-CD4 antibody.

(C) Frequency of Tfh cells within the pancreatic lymph nodes (pLN) of anti-PD-1 (n=8) compared to isotype (Iso, n=7) treated mice.

(D) Frequency of chemokine receptor CXCR6 expression on Tfh (ICOS^{+} PD1^{hi} CXCR5^{+}) CD4^{+} T cells in the pancreatic lymph node (pLN) of isotype (Iso, n=5) or anti-PD-1 (n=4) treated mice by flow cytometry.

(E) Quantification of CXCR6 expression on islet-infiltrating IL-21+ IFNγ+ Tfh cells (CD4^{+}ICOS^{+} PD1^{hi} CXCR5^{+}) in Iso (n=5) or anti-PD-1 (n=6) treated mice.

Comparison by Log Rank test (A), Welch's t test (D), or two-way ANOVA (E). *p<0.05, **p<0.01.

Supplemental Figure 2

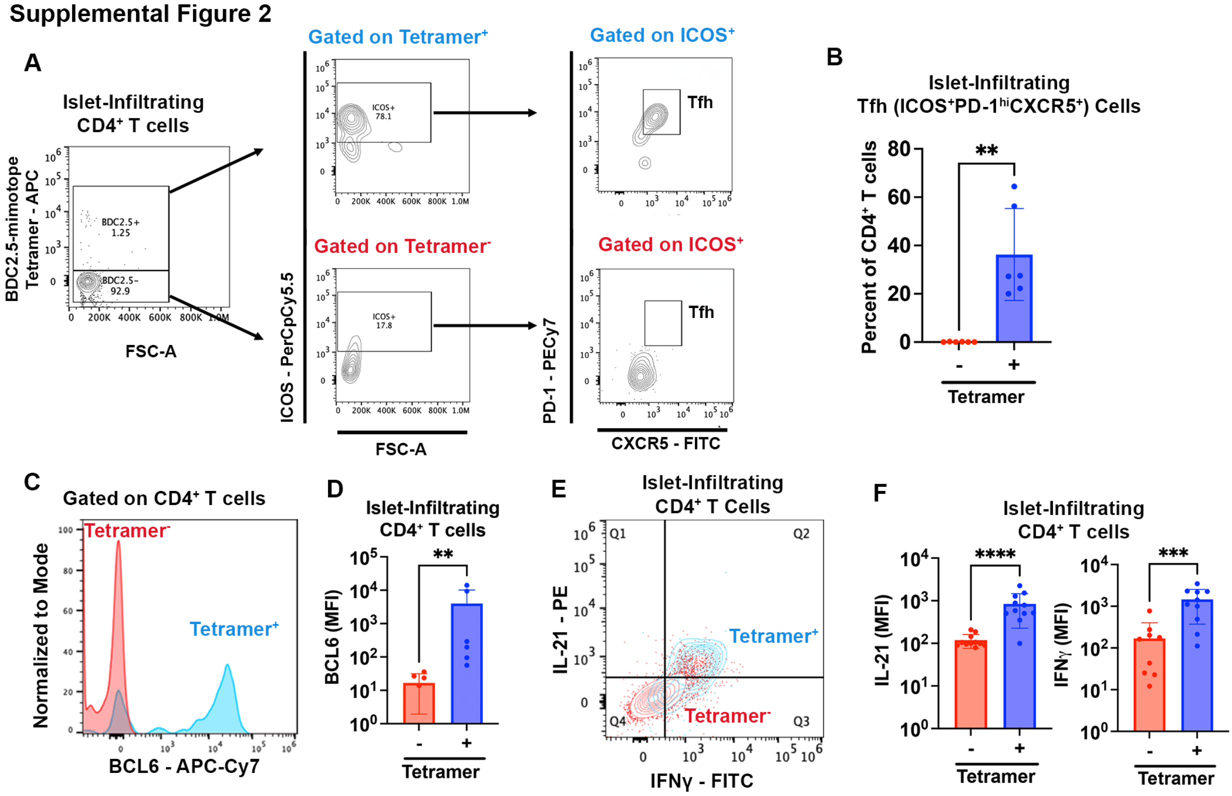

Supplemental Figure 2. BDC2.5-mimotope tetramer staining reveals antigen-specific Tfh CD4^{+} T cells within pancreatic islets of ICI-treated mice.

(A) Representative flow cytometry plots of BDC2.5-mimotope tetramer^{+} CD4^{+} T cells within the pancreatic islets of anti-PD-1 treated mice showing gating for putative T follicular helper (Tfh) cell surface markers.

(B) Frequency of Tfh (ICOS^{+} PD-1^{hi} CXCR5^{+}) cells among tetramer^{+} versus tetramer^{-} CD4^{+} T cells within pancreatic islets of anti-PD-1 treated mice.

(C) Representative histogram of Bcl6 staining among tetramer^{+} versus tetramer^{-} CD4^{+} T cells within pancreatic islets.

(D) Comparison of Bcl6 expression among tetramer^{+} versus tetramer^{-} CD4^{+} T cells within pancreatic islets in anti-PD-1 treated mice.

(E) Representative flow plot of IL-21 and IFNγ expression of tetramer^{+} and tetramer^{-} CD44^{+} CD4^{+} T cells within pancreatic islets from anti-PD-1 treated mice.

(F) Comparison of IL-21 and IFNγ expression among tetramer^{+} versus tetramer^{-} CD4^{+} T cells within pancreatic islets in anti-PD-1 treated mice.

Absolute cell counts and frequencies of islet-infiltrating cell types were determined by flow cytometry. Each point represents data from one animal and data are presented as mean±SD. Comparisons by Mann-Whitney test (B, D, F). **p<0.01; ***p<0.001, ****p<0.0001. FSC, forward scatter; PerCP-Cy5.5, peridinin chlorophyll protein-cyanine 5; PECy-7, phycoerythrin-cyanine 7; FITC, fluorescein isothiocyanate; APC, allophycocyanin; PE, phycoerythrin; FITC, fluorescein isothiocyanate; APC-Cy7, allophycocyanin-cyanine 7.

Supplemental Figure 3

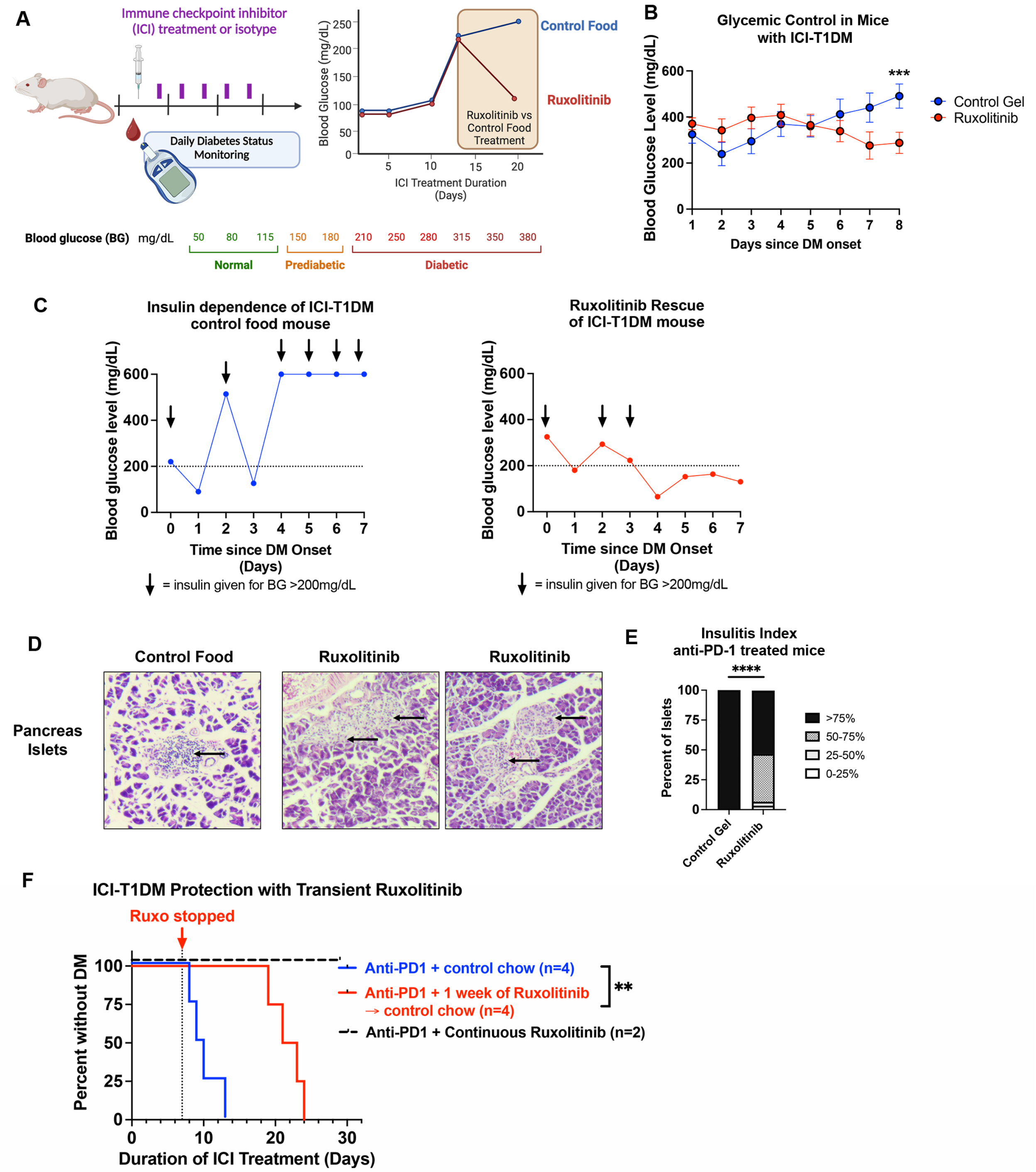

Supplemental Figure 3. Ruxolitinib improves glycemic control and reduces insulitis in ICI-T1DM even after onset of overt disease.

(A) Schematic of ruxolitinib reversal trial in mice with ICI-T1DM. Mice were treated with anti-PD-1 twice weekly and monitored for development of hyperglycemia (blood glucose >200mg/dL) daily. Once mice developed hyperglycemia, they were randomized to treatment with JAKi ruxolitinib or control food.

(B) Comparison of glycemic control mice with ICI-T1DM treated with ruxolitinib (n=12 females) or control food gel (n=12 females). Data are presented as mean±SD.
(C) Blood glucose and insulin requirements in a mouse with ICI-T1DM given control food (left) compared to a mouse given ruxolitinib (right) after onset of hyperglycemia. Data combined from three independent experiments.
(D) Representative hematoxylin and eosin staining of pancreas histology showing islets of Langerhans after 7 days of ruxolitinib therapy or control chow.
(E) Insulitis index of pancreas islet histology of mice with ICI-T1DM given ruxolitinib for 7 days or control food gel after onset of overt diabetes.
(F) DM incidence in anti-PD-1 treated mice given transient ruxolitinib therapy for 7 days then transitioned to control chow (red, 4 females). Anti-PD-1 treated mice given control chow throughout (blue, 4 females) or continued ruxolitinib (black, 2 females) were evaluated in parallel.
Comparisons by unpaired Student's t test on day eight (B), Fisher's exact test (E), or Log-Rank test (F); $^{**}p<0.01$, $^{***}p<0.001$.

Supplemental Figure 4

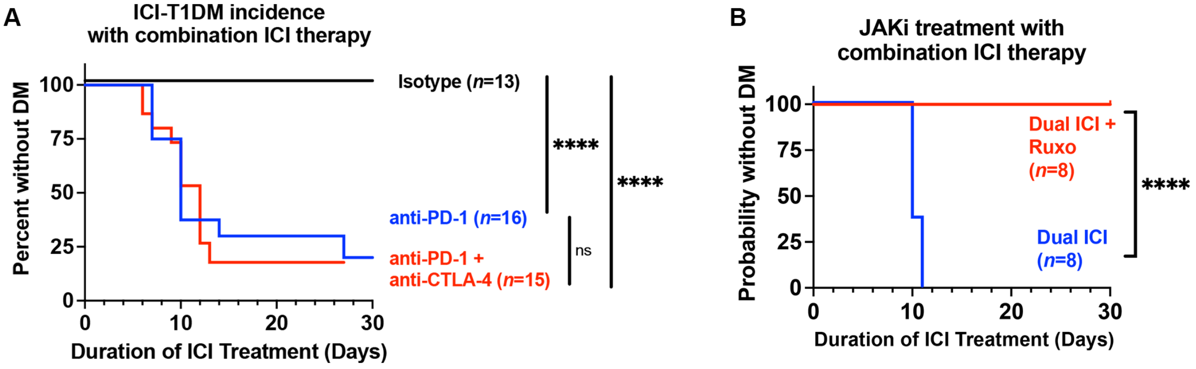

Supplemental Figure 4. Ruxolitinib prevents immune checkpoint inhibitor (ICI) autoimmune diabetes mellitus during combination ICI therapy.

(A) Incidence of autoimmune diabetes mellitus (DM) in NOD mice treated with isotype, anti-PD-1 (data reshown from Fig. 2B), and combination anti-PD-1 + anti-CTLA-4.

(B) ICI-T1DM incidence in NOD mice treated with isotype or Dual ICI (anti-PD-1 + anti-CTLA-4) in combination with ruxolitinib (1g/kg daily) or control food.

Comparisons by Log-Rank test (A, B). ****p<0.0001.
